# Notch signaling induces EMT in OSCC cell lines in a hypoxic environment

**DOI:** 10.3892/ol.2013.1549

**Published:** 2013-08-28

**Authors:** TAKAYUKI ISHIDA, HIROSHI HIJIOKA, KENICHI KUME, AKIHIKO MIYAWAKI, NORIFUMI NAKAMURA

**Affiliations:** 1 Department of Oral and Maxillofacial Surgery, Graduate School of Medical and Dental Sciences, Kagoshima University, Kagoshima 890-8520, Japan; 2 Department of Clinical Epidemiology, Institute of Industrial Ecological Sciences, University of Occupational and Environmental Health, Yahata-nishi-ku, Kitakyushu 807-8555, Japan

**Keywords:** notch, EMT, hypoxia, oral squamous cell carcinoma

## Abstract

Epithelial-mesenchymal transition (EMT) is an early step in the acquisition of invasiveness by malignant tumors. It has been clarified that the tumor microenvironment affects malignancy in a number of different carcinomas, in particular, that a hypoxic environment induces EMT. Activation of Notch signaling induces EMT, but it remains unclear how the Notch pathway is involved in oral squamous cell carcinoma (OSCC) under hypoxia. Three OSCC cell lines were cultured for examination under hypoxic (1% O_2_) and normoxic (21% O_2_) conditions. Expression of E-cadherin was investigated as a hallmark of EMT by immunohistochemical examination. Cell motility and invasion were examined by wound-healing and invasion assays, respectively. The expression of Notch pathway molecules was analyzed by qPCR. Hypoxia increased the mRNA expression of Notch receptors, ligands and target genes, and Snail. Hypoxia decreased the expression of E-cadherin, and increased the motility and invasiveness of OSCC cell lines. γ-secretase inhibitor, a Notch-specific inhibitor, prevented these effects caused by h-ypoxia. These findings suggest that hypoxia induces EMT in OSCC cell lines via activation of Notch signaling, and inhibition of the Notch signaling pathway to suppress EMT may be a useful approach for the treatment of OSCC.

## Introduction

Squamous cell carcinoma is the most common type of malignant neoplasm of the oral cavity. Due to the extent of recent investigation of its pathogenesis and management, the five-year survival rate for patients with oral squamous cell carcinoma (OSCC) has marginally improved within the last 15 years, yet it remains as low as 60% (1). The presence of cervical lymph node metastasis is the foremost reliable, adverse prognostic factor in patients with OSCC. The presence of metastatic spread to the regional lymph nodes correlates strongly with a poor overall prognosis, an increased risk of distant metastasis and a reduction in the five-year survival rate by ~50% (2).

Epithelial-mesenchymal transition (EMT) is a key step toward cancer metastasis. EMT is marked the by loss of epithelial characteristics (such as cell polarity and cell-cell junctions) and the gain of mesenchymal characteristics (including fibroblastic spindle-shaped morphology and increased motility). It has been proposed that EMT may provide a link between cancer metastasis and stem cell properties (3).

The tumor microenvironment appears to play a prominent role in affecting EMT changes. For example, the E-cadherin transcriptional repressor, TWIST, is positively regulated by hypoxia-induced factor 1α (HIF-1α) (4). In a previous study, a number of different types of cancer cells were exposed to carefully controlled hypoxic conditions and investigated for EMT changes (5).

Notch signaling influences a variety of cellular processes, including cell fate specification, proliferation, differentiation, apoptosis and the maintenance of stem cells (6). To date, four vertebrate Notch genes have been identified: Notch1-4. In addition, five ligands, Dll1, Dll3, Dll4 and Jagged1/2, have been identified in mammals. Aberrant Notch signaling is detected in a range of human tumor types. Upregulated expression of Notch receptors and their ligands has been identified in cervical, lung, colon, renal and pancreatic cancer, as well as in acute myeloid leukemia and Hodgkin’s and large-cell lymphomas (7,8).

Certain studies have revealed links between hypoxia and activation of Notch in solid tumors (9,10). Low oxygen content has been observed to potentiate Notch signaling in melanocytes through stabilization of HIF-1α (11). In addition, Notch signaling and Wnt pathways have been demonstrated to be required for conversion of the hypoxic stimulus into EMT, increased motility and invasiveness (12).

However, the molecular mechanisms by which hypoxia affects EMT and the Notch signaling pathway, in order to increase the invasiveness and metastatic potential of OSCC, are unclear. In this study, hypoxia potentiated EMT, which was induced by Notch signal activation, thus enhancing motility and invasiveness in human OSCC cell lines.

## Materials and methods

### Cell culture

Human OSCC cell lines, HSC-2, HSC-4 and Ca99-2, were purchased from Health Science Research Resources Bank (Osaka, Japan). Cell lines were cultured in Minimum Essential Medium Eagle (Sigma-Aldrich, St. Louis, MO, USA), supplemented with 10% fetal bovine serum (CCB, Nichirei Bioscience, Tokyo, Japan), penicillin (100 U/ml), streptomycin (100 μg/ml) and amphotericin B (0.25 μg/ml) (Invitrogen, Carlsbad, CA, USA). The cells were placed under hypoxia (1% O_2_) or normoxia (21% O_2_) for 24 h. Notch activity was blocked by 5 μM DAPT, a γ-secretase inhibitor (GSI; Calbiochem, Basel, Switzerland) in immunohistochemical, wound-healing and invasion assays.

### qPCR

Total RNAs were isolated using TRIzol (Invitrogen), and cDNAs were synthesized with a High-Capacity cDNA Reverse Transcription kit (pplied Biosystems, Foster City, CA, USA) from 1 μg of total RNA. qPCR was performed with the cDNA samples and 2X SYBR-Green PCR master mix (PE Applied Biosystems), using a Step One™ Real-Time PCR system (Applied Biosystems). The formation of the PCR product was monitored using SYBR-Green (Applied Biosystems). All samples were amplified in duplicate. The relative changes in the levels of the transcripts in each sample were determined by normalization with the β-actin mRNA levels. The sequences of the primers used in the real-time PCR are listed in Table I.

### Immunohistochemical examination

Cells were fixed with 4% paraformaldehyde (Sigma-Aldrich) in phosphate buffered saline (PBS) and subjected to immunofluorescence staining. Cells were blocked with PBS containing 10% fetal calf serum and 0.1% Triton X-100 (Sigma-Aldrich). The primary rabbit polyclonal antibody used was anti-E-Cadherin (diluted 1:200; Calbiochem, Basel, Switzerland). A fluorescent rhodamine-conjugated donkey anti-rabbit IgG antibody (diluted 1:200; Chemicon, Temecula, CA, USA) was used as the secondary antibody.

### Wound-healing assay

Cell migration was analyzed by a scratch wound-healing assay. Cells were grown to confluence and a scratch wound was made in the monolayer by dragging a pipette tip across it. Detached cells were washed away with PBS, and fresh medium was used for culture under hypoxia or normoxia. Photomicrographs of three areas were simultaneously captured under a phase contrast microscope (CKX41; Olympus, Tokyo, Japan), and migrated cells were counted in three scratched areas at magnification, ×400.

### Invasion assay

Cell invasion was analyzed using the BD BioCoat™ Matrigel™ Invasion Chamber (BD, Franklin Lakes, NJ, USA), according to the manufacturer’s instructions. Individual cells were plated in the upper insert, at a density of 1.5×10^5^ cells/ml for a 24-well chamber, in serum-free medium. Medium containing 10% fetal bovine serum as a chemoattractant was added to the well. The cells were placed under hypoxia (1% O_2_) or normoxia (21% O_2_) for 24 and 48 h. Invaded cells were stained by Diff-Quik kit (Sysmex, Kobe, Japan) according to the manufacturer’s instructions. Invaded cells were counted in three suitable areas by stereoscopic microscope (BH-2; Olympus) at magnification, ×400.’

### Statistical analyses

Data are presented as mean ± standard deviation. Differences were analyzed to determine their statistical significance using Student’s t-test. P<0.05 was considered to indicate a statistically significant difference.

## Results

### Expression of Notch signaling molecules and Snail mRNA is upregulated by hypoxia

**Figure 1:**
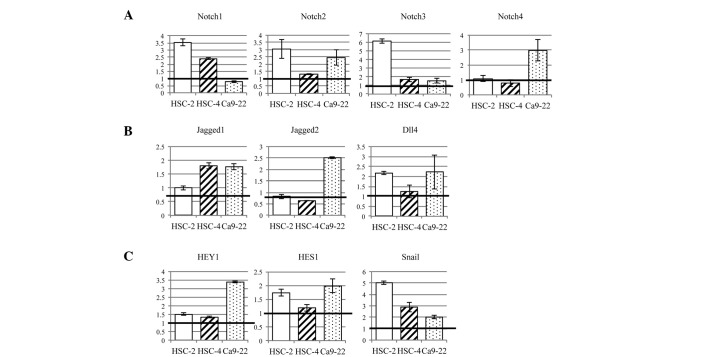
Expression of Notch signaling pathway genes was increased by hypoxia, compared with the levels under normoxia, in OSCC cell lines. (A) Notch receptors were upregulated in all three OSCC cell lines by hypoxia; in particular, HSC-2 cells showed a marked increase in Notch1-3 expression. (B) Three Notch ligands, Jagged1/2 and Dll4, were examined. These ligands were increased in OSCC cell lines by hypoxia. (C) Notch target genes, HEY1 and HES1, were upregulated by hypoxia. Snail, a marker of EMT, was increased by hypoxia in all OSCC cell lines. OSCC, oral squamous cell carcinoma; EMT, epithelial-mesenchymal transition.

To analyze whether Notch receptor activity was promoted by hypoxia in OSCC cell lines, Notch1-4 mRNA expression was investigated using qPCR (Fig. 1A). Notch1 mRNA expression was upregulated in HSC-2 cells (3.52-fold) and HSC-4 cells (2.45-fold) under hypoxia, compared with the levels under normoxia. Notch2 was upregulated in HSC-2 cells (3.04-fold) and Ca9-22 cells (2.45-fold) under hypoxia. Notch3 mRNA expression was upregulated in HSC-2 cells (6.13-fold), and Notch-4 was upregulated in Ca9-22 cells (2.97-fold). These results indicated that hypoxia increased the expression level of Notch receptor mRNA in OSCC cell lines.

We examined three Notch ligands, Jagged1/2 and Dll4 (Fig. 1B). Expression of Jagged1 mRNA was increased in HSC-4 cells (1.80-fold) and Ca9-22 cells (1.76-fold) by hypoxia. Jagged2 was increased in Ca9-22 cells (2.51-fold). Dll4 was increased in HSC-2 cells (2.17-fold) and Ca9-22 cells (2.23-fold). Thus, hypoxia promoted Notch ligand expression in OSCC cell lines.

The expression of Notch target genes, HEY1 and HES1, was increased in all three OSCC cell lines (1.19- to 3.38-fold) (Fig. 1C). In particular, HEY1 and HES1 were upregulated, 1.98- and 3.38-fold in Ca9-22 cells and 1.73- and 1.51-fold in HSC-2 cells, respectively, compared with the levels under normoxia. In HSC-4 cells, HES1 and HEY1 were not markedly affected by hypoxia (1.33- and 1.19-fold, respectively).

Snail, an E-cadherin repressor, was increased in all three OSCC cell lines by hypoxia (2.01- to 5.03-fold) (Fig. 1C). This indicated that a hypoxic environment promoted EMT through increased expression of Snail.

### Hypoxia decreases expression of E-cadherin via activation of Notch signaling

**Figure 2:**
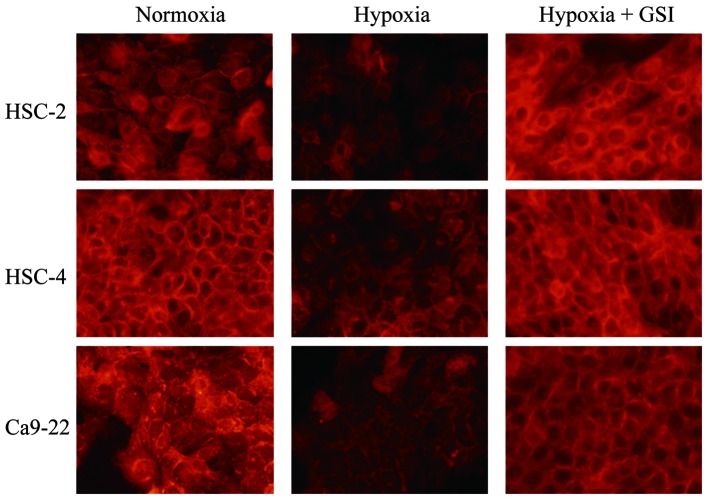
Expression of E-cadherin in OSCC cells cultured under normoxia and hypoxia, with or without GSI. Immunohistochemical examination revealed that hypoxia suppressed the expression of E-cadherin in all three OSCC cell lines. GSI (5 μM) inhibited the suppression of E-cadherin expression by hypoxia. OSCC, oral squamous cell carcinoma; GSI, γ-secretase inhibitor.

The expression of E-cadherin was examined in a 24-h culture under hypoxia or normoxia. In all three OSCC cell lines, E-cadherin expression decreased upon culture under hypoxia compared with that under normoxia (Fig. 2), indicating that hypoxia induced EMT in OSCC cell lines. Notch signaling-induced EMT in a hypoxic environment was subsequently examined. GSI, a Notch inhibitor, prevented downregulation of E-cadherin by hypoxia in all OSCC cell lines. These findings indicated that hypoxia activated the Notch signaling pathway to induce EMT in OSCC cell lines.

### Hypoxia enhances cell motility and invasion via Notch signaling

**Figure 3:**
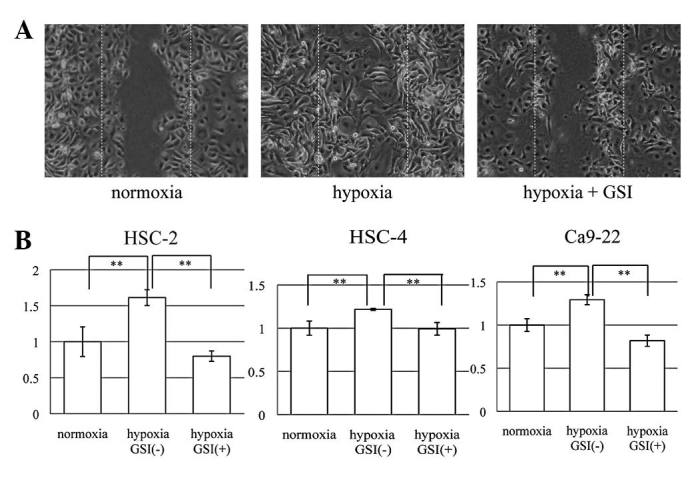
(A) Scratch wound healing assay of HSC-2 cells cultured under hypoxia and treated with or without GSI, compared with those cultured under normoxia as a control. (B) The number of migratory cells in the scratched area was counted in three areas. Hypoxia significantly increased the migrated cell ratio compared with that of normoxia in all cell lines. GSI (5 μM) significantly prevented the promotion of cell migration by hypoxia in all cell lines. These findings suggested that hypoxia enhanced the cell migration of OSCC cell lines through Notch signaling pathway activation. ^**^P<0.01. GSI, γ-secretase inhibitor; OSCC, oral squamous cell carcinoma.

**Figure 4:**
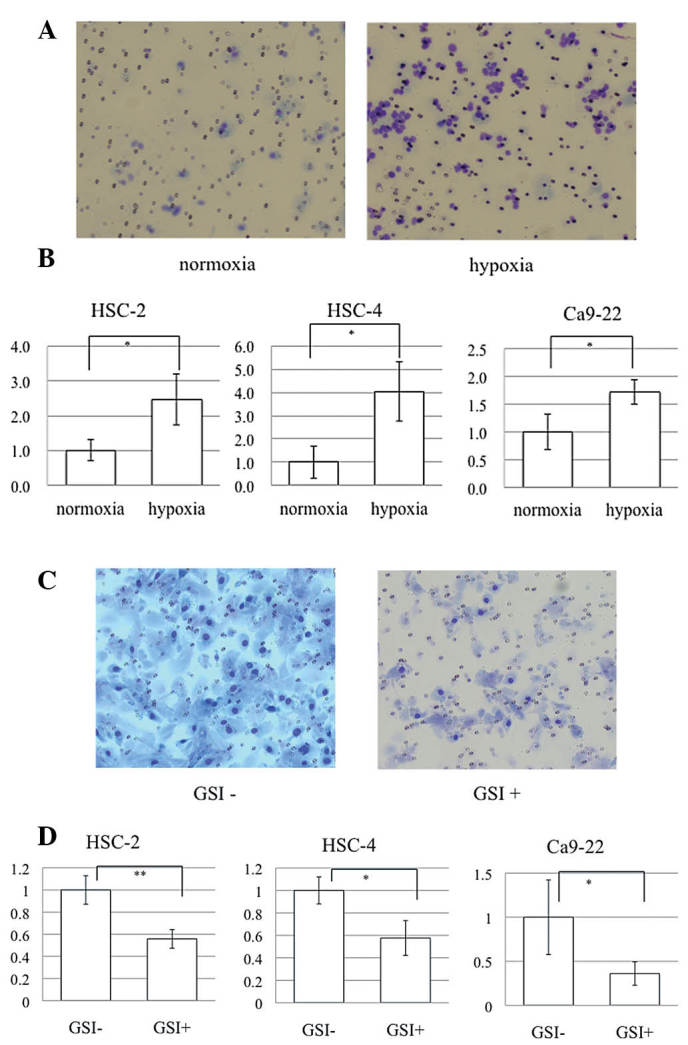
(A) Following culture under hypoxia or normoxia, invasive cells were stained using Diff-Quik for counting. (B) The ratio of invaded cells was significantly increased by hypoxia compared with that under normoxia in HSC-2, HSC-4 and Ca9-22 cells (^*^P<0.05). (C) To analyze whether hypoxia enhanced cell invasion through the Notch signaling pathway, cells were cultured with or without 5 μM GSI under hypoxia for 48 h. (D) GSI prevented cell invasion significantly in OSCC cell lines (^*^P<0.05 and ^**^P<0.01). These findings suggested that hypoxia promoted the invasiveness of OSCC cell lines through the Notch signaling pathway. GSI, γ-secretase inhibitor; OSCC, oral squamous cell carcinoma.

To study cell motility, we used a scratch wound healing assay (Fig. 3A), in which the extent of migration of cells into the scratched areas was counted at three points. Quantification of relative closure of the scratch wound was undertaken and compared with that of the control. All three OSCC cell lines showed significantly increased cell motility due to hypoxia (P<0.01), and GSI significantly prevented this effect (P<0.01) (Fig. 3B). These results indicated that hypoxia upregulated cell motility through activation of the Notch signaling pathway in OSCC cell lines. To examine whether a hypoxic environment promoted the invasiveness of OSCC cell lines, we conducted an invasion assay cultured under hypoxia or normoxia for 24 h (Fig. 4A). All three OSCC cell lines showed an increased cell invasion ratio under hypoxia, compared with that under normoxia (Fig. 4B). The number of invasive cells under hypoxia, compared with that under normoxia, was increased 2.47-fold in HSC-2 cells (P<0.05), 4.05-fold in HSC-4 cells (P<0.05) and 1.71-fold in Ca9-22 cells (P<0.05). Subsequently, it was examined whether hypoxia promoted the increased invasiveness via the Notch signaling pathway. Cells were cultured under hypoxia, with or without 5 μM GSI, for 48 h (Fig. 4C). All three OSCC cell lines showed a decreased cell invasion ratio with GSI. The number of invasive cells with GSI, compared with those without GSI, was decreased 0.56-fold in HSC-2 cells (P<0.01), 0.58-fold in HSC-4 cells (P<0.05) and 0.36-fold in Ca9-22 cells (P<0.05). These results indicated that hypoxia enhanced tumor invasiveness through Notch pathway activation in OSCC cell lines.

## Discussion

Tumor metastasis entails a series of distinct, sequential steps, involving local tumor growth, invasion by transmigration through basement membranes and non-tumor host tissue, intravasation into blood vessels, dissemination and survival in the bloodstream, extravasation and re-establishment at distant sites (13). EMT also occurs during the early stages of carcinogenesis to bypass oncogene-induced senescence (14). Several studies have demonstrated the expression of EMT-specific genes at the invasive front of primary tumors, thus providing evidence that EMT may constitute a critical prerequisite for primary tumor cells to break through the basal lamina into non-involved adjacent host tissue (15,16). Clinical evidence has indicated that tumor hypoxia is a poor prognostic factor for patient outcome (17–19). A number of different types of cancer cells were observed to respond to hypoxic exposure within 72 h by classic EMT changes (fibroblastoid phenotype, Snail and β-catenin nuclear translocation, and changes in E-cadherin), and they exhibited increased migration and invasiveness (20).

In the present study, hypoxia downregulated the expression of E-cadherin in three OSCC cell lines, as shown by immunohistochemical examination. Additionally, OSCC cell migration and invasion were also facilitated by culture in 1% O_2_, compared with the levels in 21% O_2_. These results suggest that a hypoxic environment induces EMT and an aggressive phenotype in OSCC cell lines.

The Notch signaling pathway exhibits different downstream effects depending on which sub-site or microenvironment it is influencing (21). Recently, two studies hypothesized that Notch1 has a tumor-suppressive function in head and neck squamous cell carcinoma (HNSCC) (22,23). The results of the present study indicated that Notch signaling acts as an oncogenic factor under hypoxia in OSCC cell lines. Previously, it was suggested that inhibition of the Notch pathway suppresses OSCC growth (24). Notch1 has been implicated as a downstream effector of oncogenic Ras in human mammary tumorigenesis (25). Patients with tumors expressing high levels of Jagged1 protein exhibited a poorer outcome than those with tumors expressing low levels of this protein in breast cancer (26,27), HNSCC (28) and tongue squamous cell carcinoma (29). The critical role of Notch signaling in EMT has been demonstrated by blocking Notch signaling, via knockdown of either HEY1 or Jagged1 expression (30), or by γ-secretase inhibitor attenuation of EMT (31). The present study demonstrated that hypoxia decreased the expression of E-cadherin and increased cell motility and invasion, but GSI inhibited these effects. These results indicate that hypoxia induced EMT through upregulation of the Notch signaling pathway as a form of oncogenic activation in the OSCC cell lines.

The expression of mRNA associated with Notch signaling in OSCCs cultured in 1% O_2_ or 21% O_2_ for 24 h was examined using qPCR. All three OSCC cell lines showed upregulation of the mRNA levels of Notch receptors, ligands and target genes under hypoxia, compared with the levels under normoxia, indicating that the Notch signaling was enhanced by the hypoxic stimulus in the OSCC cell lines. In addition, culture of lung (9) and ovarian (31) cancer cells under hypoxia has been observed to increase Notch signaling pathway activation. Low oxygen content has also been demonstrated to potentiate Notch signaling in melanocytes through stabilization of HIF-1α (11). HIF-1α is a transcription factor that activates transcription of genes involved in anaerobic metabolism, angiogenesis, survival, invasion, metastasis and treatment resistance in tumor cells, thus promoting cellular adaptation and survival under hypoxic conditions. HIF-1α is critical in hypoxia-activated gene expression (32). Stabilization and activation of the HIF-1α transcription complex also correlates with tumor metastasis and poor prognosis in patients with cancer (33–35).

Upregulation of Snail correlates with metastasis and poor prognosis, whereas silencing of Snail is critical for reducing tumor growth and invasiveness (36,37). Hypoxia may attenuate the expression of E-cadherin, via activation of the lysyl oxidase (LOX)-Snail pathway, to promote tumor invasion and metastasis. This indicates that hypoxia-induced LOX and HIFs are important factors that regulate tumor microenvironments to favor metastasis (38,39). Although the present study demonstrated that the level of Snail mRNA was increased by hypoxia in these OSCC cell lines, no clear association between Notch signaling pathway activation and Snail expression was observed. However, previous studies have indicated that Snail expression is directly regulated by the Notch signaling pathway (31,40). Further investigation is necessary to clarify this issue in OSCC.

The current study identified that hypoxia promoted cell motility and invasion, decreased the expression of E-cadherin and upregulated the Notch signaling pathway, indicating that hypoxia induced EMT in the OSCC cell lines. GSI inhibited this upregulation of cell motility and invasion, and the decrease in expression of E-cadherin under hypoxia. These results suggest that hypoxia induced EMT through the upregulation of Notch signaling activation. Elucidation of the mechanism of EMT through activation of the Notch signaling pathway may provide novel molecular targets and contribute to improving the prognosis of patients with OSCC.

## Figures and Tables

**Table I:** PCR primer sequences.

Target molecules	Primer sequence
Notch1	Forward: 5′-CAATGTGGATGCCGCAGTTGTG-3′ Reverse: 5′-CCATCCTGGGACTTCTTCCT-3′
Notch2	Forward: 5′-AAAAATGGGGCCAACCGAGAC-3′ Reverse: 5′-TTCATCCAGAAGGCGCACAA-3′
Notch3	Forward: 5′-TCTTGCTGCTGGTCATTCTC-3′ Reverse: 5′-TGCCTCATCCTCTTCAGTTG-3′
Notch4	Forward: 5′-CACTGAGCCAAGGCATAGAC-3′ Reverse: 5′-ATCTCCACCTCACACCACTG-3′
Jagged1	Forward: 5′-CGGGATTTGGTTAATGGTTATC-3′ Reverse: 5′-ATAGTCACTGGCACGGTTGTAGCAC-3′
Dll4	Forward: 5′-TGACCACTTCGGCCACTATG-3′ Reverse: 5′-AGTTGGAGCCGGTGAAGTTG-3′
HES1	Forward: 5′-AGGCGGACATTCTGGAAATG-3′ Reverse: 5′-CGGTACTTCCCCAGCACACTT-3′
HEY1	Forward: 5′-CGAGGTGGAGAAGGAGAGTG-3′ Reverse: 5′-CTGGGTACCAGCCTTCTCAG-3′
Snail	Forward: 5′-CATCCTTCTCACTGCCATGGA-3′ Reverse: 5′-AGGCAGAGGACACAGAACCAGA-3′
β-actin	Forward: 5′-AAGAGATGGCCACGGCTG-3′ Reverse: 5′-GAACCGCTCATTGCCAATG-3′
